# Intradural calcifying pseudoneoplasm of the neuraxis in the lumbosacral canal: Two case reports and review of the literature

**DOI:** 10.1016/j.ijscr.2025.111966

**Published:** 2025-09-21

**Authors:** Shuo Han, Guang-Ming Wang, Da-Wei Dai, Dan-Feng Zhang

**Affiliations:** a Department of Neurosurgery, Shanghai Changzheng Hospital, Shanghai, 200003, China; b Department of Neurosurgery, Shanghai Renji Hospital, Shanghai, 200127, China

**Keywords:** Calcifying pseudoneoplasm, Spine, Surgery, Case report

## Abstract

**Background:**

Calcifying pseudoneoplasms of the neuraxis (CAPNON) are benign and slowly growing fibro-osseous lesions of the nervous system.

**Methods:**

We report two rare cases of spinal CAPNON and provide a literature review.

**Results:**

A 33-year-old woman with back pain underwent lumbar magnetic resonance imaging (MRI), revealing a large intradural mass (1.5 × 0.9 × 10.6cm^3^) at L2-S1. Postoperative MRI scan performed 3 years after surgery confirmed no recurrence. A 64-year-old woman with lower limb numbness and gait instability underwent lumbar MRI, revealing an L3 intradural mass (1.1 × 0.3 × 1.6cm^3^). Lower limb numbness were resolved after surgery during 1 year follow-up.

**Conclusion:**

Accurate recognition of CAPNON is essential to guide appropriate surgical intervention due to its favorable prognosis. In these situations, complete resection and radiological follow-up are highly recommended.

## Highlights

• The first case of 10.6 cm intradural CAPNON in the lumbosacral canal spanning five spinal segments and we review the literature briefly.
• Current recommendations for the management of spinal CAPNON are discussed.
• Differentiation of spinal CAPNON from true tumors is critical to avoid unnecessary treatments due to its favorable prognosis.

## 1 Introduction

Calcifying pseudoneoplasms of the neuraxis (CAPNON) are heavily calcified parenchymal lesions upon histopathology involving the central nervous system. The etiology of CAPNON is hypothesized to be reactive to trauma, inflammation, or hemorrhage. CAPNON rarely occurs in the spine and is seldom reported in the literature since the first description by Rhodes and Davis in 1978. To our knowledge, no previously reported CAPNON has exceeded three spinal segments in length. We herein describe the first case of an intradural CAPNON in the lumbosacral canal, measuring approximately 14 cm^3^ and spanning five spinal segments. This case report has been reported in line with the SCARE checklist [1].

## 2 Clinical presentation

### 2.1 Case 1

**Fig. 1:**
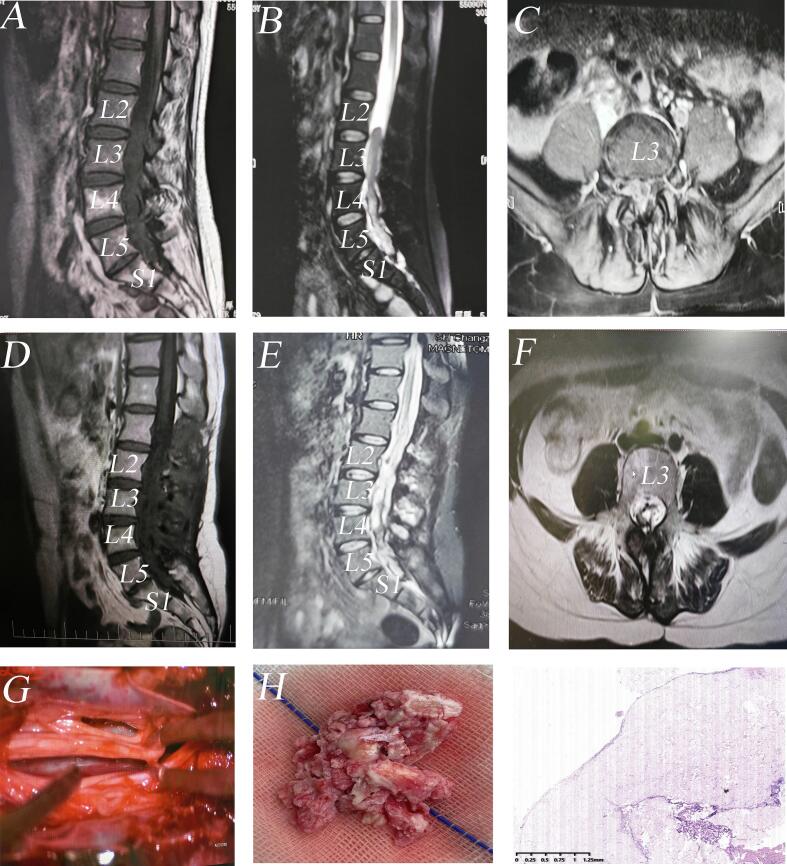
(A) Pre-operative sagittal T1-weighted images demonstrating a hypointense intradural mass at the L2-S1 levels.(B) Sagittal T2-weighted images revealing a mixed-signal lesion. (C) Post-contrast sequences showing the mass within the right lateral aspect of the spinal canal. (D) Post-operative sagittal T1-weighted images showing the extradural mass was completely removed.(E) Axial T2-weighted images confirming the remaining cavity in the surgery area. (F) Post-contrast sequences illustrating no enhancement after surgery. (G) Intraoperative images demonstrating the lesion being completely resected along with its external capsule for pathological examination. (H)Tumor specimen showing prominent calcification and firm consistency, which complicated resection. (I) Immunohistochemical EMA showing positive expression of some spindle-shaped stromal cells, suggesting that the tumor may originate from fibroblasts in the arachnoid or choroidal plexus stroma.

A 33-year-old woman with a 5-year history of progressively worsening low back pain without myelopathy/radiculopathy. On examination, her vital signs were stable. A neurological examination revealed normal muscle reflexes and negative lasegue's test. A renal computed tomographic (CT) scan incidentally identified a hyperdense lesion in the lumbosacral canal. Lumbar magnetic resonance imaging (MRI) revealed a large intradural mass (1.5 × 0.9 × 10.6 cm^3^) at L2-S1. The lesion exhibited hypointensity on T1-weighted images, mixed signal intensity on T2-weighted images and no contrast enhancement (Fig.1). We obtained consent from the patient, and then surgical resection was performed via a posterior midline approach. After partial removal of L2-S1 lamina using high-speed burr without foraminotomy and facetectomy, a stonelike mass was found in the interlayer between the connective tissue membrane and the dura while being noted to be firmly adherent to the dura mater and roots, encased in a layer of connective tissue membrane resembling the dura mater in appearance. The lesion was meticulously dissected and completely resected for pathological examination. Subsequently, a watertight dural closure was obtained using a combination of sutures and fibrin sealant, with laminoplasty using titanium miniplates alone. Pathological examination showed amorphous calcifying masses with osseous metaplasia in a fibrovascular stroma. Immunohistochemistry revealed focal EMA positivity in spindle-formed stromal cells, suggesting arachnoid/choroidal plexus fibroblastic origin. Postoperatively, the patient's pain resolved completely with no recurrence at 3-year follow-up MRI.

### 2.2 Case 2

**Fig. 2:**
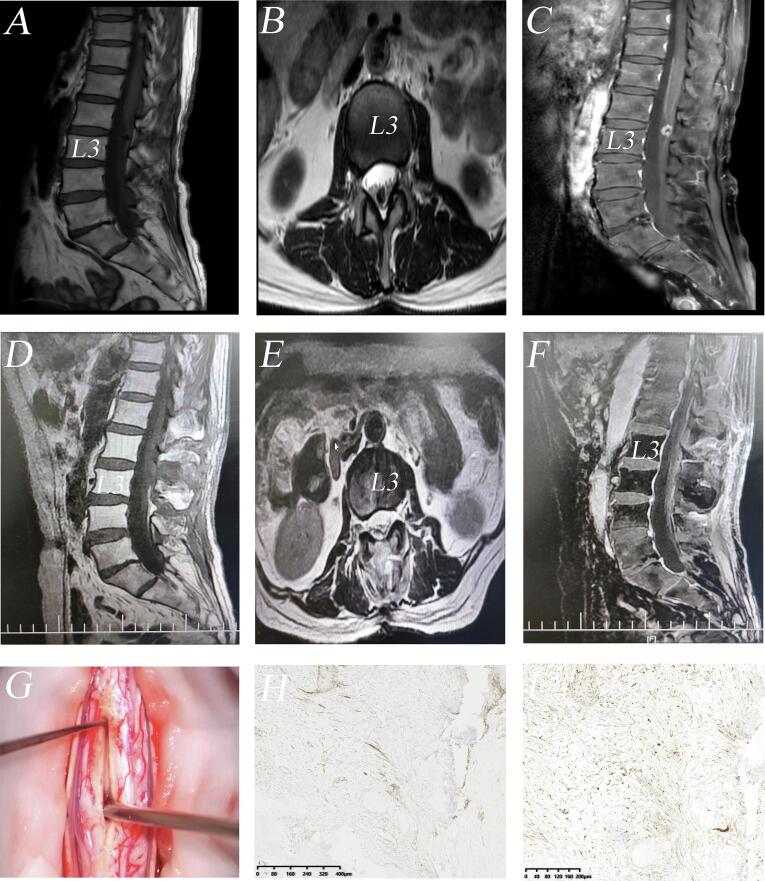
(A) Pre-operative sagittal T1-weighted images revealing a hypointense intradural mass at the L3 level. (B) Axial T2-weighted images showing the mass within the left posterolateral aspect of the spinal canal. (C) Post-contrast sequences demonstrating peripheral enhancement. (D) Post-operative sagittal T1-weighted images confirming the extradural mass was completely removed. (E) Axial T2-weighted images showing no remaining mass. (F) Post-contrast sequences revealing no postoperative enhancement. (G) Intraoperative images demonstrating resection via a posterior approach with a sharp scalpel blade through microscopic manipulation. (H) EMA immunostaining highlighting positive expression in interstitial spindle cells. (I) CD68 (KP1) immunostaining showing positivity in interstitial histiocytes.

A 64-year-old woman presented with a two-month history of bilateral lower limb numbness and unsteady gait. Vital signs (BP, HR, RR, Spo_2_) were all within normal limits. Bilateral leg were elevated over 70° without pain. Symmetric reflexes were graded 2+. Lumbar MRI demonstrated a T1 hypointense, T2 heterogeneously intense intradural mass (1.1 × 0.3 × 1.6cm^3^) at L3, without contrast enhancement. Under general anesthesia, the patient was placed in the prone position. After partial removal of L3 lamina without foraminotomy and facetectomy, gross total resection was achieved through microscopic manipulation. The hypovascular mass was noted intraoperatively to be hard, greyish-white, and non-adherent to the dura or roots. Finally, we reattached the lamina using titanium miniplates and performed intradermic suture. Tumor specimen showed well-demarcated calcification alongside ossification, featuring palisading spindle-to-epithelioid cells, fibrous stroma, and multinucleated giant cells. Immunostaining revealed epithelial membrane antigen (EMA) positivity in spindle cells and CD68 (KP1) positivity in interstitial histiocytes (Fig.2). Postoperatively, pain resolved completely, with no recurrence during 1 year follow-up.

## 3 Discussion

CAPNON are benign fibro-osseous lesions that mimic tumors but lack malignant potential [2]. Our literature review identified 296 potentially relevant publications. After applying inclusion criteria, only 83 histologically confirmed spinal CAPNON cases were reported (Table 1) [[2], [3], [4], [5], [6], [7], [8], [9], [10], [11], [12], [13], [14], [15], [16], [17], [18], [19], [20]]. CAPNON most frequently occurred in adults (age range: 7–90 years), with female predilection (47 females, 36 males). Yet it's worth noting that most of the lesions in the spine were in the extradural space (55 located as extradural, 17 intradural, 2 transdural) (*p* < 0.0001). Further, CAPNONs were more likely to be located in the lumbar spine (41 cases) compared to cervical (19 cases) or thoracic (16 cases) (p < 0.0001).

**Table 1:** Demographic, clinical, radiological and result of published cases of CAPNON, including our current cases.

Case	Author	Age, sex	Clinical sign	Location	Radiographic findings Size	Pathological findings	Surgical resection	Result
1	Bertoni, 1990	23, M	Back pain 5 years	T10, extradual	Calcification of the lesion on CT	HE: The granulomas were either nodular or confluent, producing a large mass with peripheral lobular configuration. Extrathelioid cells and giant cells bordered the granulomas.	ST	No recurrence at 2 mo
2		58, M	Intermittent low back pain 15 years, progressive stiffness 3 months	C2–3, extradual	Calcification of the lesion on CT		ST	Died
3		12, M	Neck stiffness and pain 1 month	C6, extradual	Calcification of the lesion on CT		ST	No recurrence at 3 years
4		32, M	Back pain many years	L4–5, extradual	Calcification of the lesion on CT		ST	No recurrence at 7 years
5		33, F	Mid Back pain 3 months	T9, extradual	Calcification of the lesion on CT		ST	No recurrence at 1 mo
6		68, F	Sciatica 5 months	L4–5, extradual	Calcification of the lesion on CT		ST	No recurrence at 1 year
7		20, F	Incidental back pain 2 month	C1–2, extradual	Calcification of the lesion on CT		ST	Died
8		56. F	Back pain 1 year	L4–5, extradual	Calcification of the lesion on CT		ST	No recurrence at 1 mo
9	Moser, 1994	68, M	Left arm radicular pain 1 month	C7-T1, extradual	Hypointense on T1w, hyperintense on T2w with minimal peripheral enhancement	None	GT	No recurrence at 2 mo
10	Smith, 1994	49, M	Left leg radicular pain 4 years	L2–3, extradual	Hypointense on T1w and T2w	GFAP(+)	ST	No recurrence at 4 mo
11	Qian, 1999	59, M	Neck pain 30 years	C1–2, extradual	N	None	GT	No recurrence at 4 years
12		49, M	Left upper limb pain and sensory loss	Clivirus region	N	None	GT	No recurrence at 90 months
13	Shrier,1999	59, M	Longstanding neck pain presented with shuffling gait and decreased sensation in the left hand	Foramen magnum	Hypointense on T1w and T2w with prominent contrast enhancement	GFAP(−),S100(−), HE:mature lamellar bone in a fibrous stroma.	GT	No recurrence at 2 years
14	Chang, 2000	60, M	Neck pain and stiffness 4 years	C2,intradural	Hypointense on T1w and T2w with contrast enhancement, increased isotope uptake on bone scintigraphy	None	ST	No recurrence at 2 years
15	Mayr, 2000	58, M	Progressive jerkiness in lower extremities and worsening back pain 1 year	T10–12, extradual	Hypointense on T1w and T2w with minimal peripheral enhancement	HE:Central calcified granular debris surronded by reactive tissue containing multinucleated giant cells	Debulking and biopsy	Non't mentioned
16		63, M	Loss of feeling in arms below the elbows and gait dysfunction 3 months	C3–4, extradual	Hypointense on T1w and T2w	HE: degenerated calcified granular material surrounded by osteoclast-like giant cells	ST	No recurrence at 5 years
17	Liccardo, 2003	40, M	Thoracic/chest pain 2 months	T8, extradual	Hypointense on T1w and T2w without contrast enhancement	HE: a fibrotic area comprising calcifications and multinucleate giant cells	GT	No recurrence at 3 years
18	Park, 2008	59, F	Neck pain and left arm radicular pain 4 months	C7-T1, extradual	Isointense on T1w and T2w with minimal peripheral enhancement	HE: basophilic plates arranded in parallel with few nuclei scattered with them	GT	No recurrence at 1 mo
19	Apostolopoulos,2009	53, M	A long history of low back pain and a few months of occasional left groin pain	L1, intradural	With contrast enhancement	S100(−). HE:proliferation of spin- dle-shaped fibroblastic cells (F) and collections of calcified but not ossified matrix	GT	A small area of numbness of the left groin
20	Tong, 2010	67, F	Chronic lower back pain and inability to walk	L4–5, extradual	Multiple calcified foci on CT	HE: calcified fibro-osseous fragments and a nearly acellular chondromyxoid matrix	Debulking and biopsy	Non't mentioned
21	Rulseh, 2011	43, F	Recurrent low back pain and radicular pain	L3, extradual	Hypointense on T1w and T2w	HE: a calcified lesion consisting of primitive bone trabeculae and islets of choroid tissue in a moderately cellular matrix, scattered psammoma bodies and a fibrous stroma	GT	No recurrence at 10 mo
22	Ozdemir,2011	53,M	Left side of face pain 1 year	Foramen magnum	With contrast enhancement	None	GT	None
23	Muccio, 2012	57, M	Low back pain 4 years	T10–11, extradual	Hypointense on T1w, T2w and STIR without contrast enhancement	HE: the abnormal tissue is com- posed of mature bone (single asterisk) encincling a granulomatous tissue made up of nodular and confluent structures.	GT	No recurrence at 2 mo
24	Kwan, 2012	48, M	Left chest and lower limb radicular pain 8 weeks	T9–10, extradual	Hypointense on T1w and T2w without contrast enhancement	None	Indomethacin	No recurrence at 4 mo
25	Naidu, 2012	43, M	Low back pain and left leg radicular pain	L4, extradual	Iso-hyperintense on T1w, mixed hypointense and hyperintense on T2w, with diffuse reticular postcontrast enhancement	HE: numerous areas of osseous metaplasia	GT	No recurrence at 2 mo
26	Nathoo,2012	44, F	Progressive left- sided flank pain 1 year	L4–5,extradural	Hypointense on T1w and T2w	HE: g concentric calcospherites (psammoma bodies) within the paucicellular, fibrosclerotic spindle cells	GT	No recurrence at 18 mo
27	Jentoft, 2012 [2]	26, F	Low back pain	L1–2,intradural	Hypointense on T2w with minimal contrast enhancement	EMA and SMA (+). S100(+): An axon bundle within the substance of the pseudoneoplasm is highlighted.	GT	No recurrence at 3 mo
28	Bartanusz, 2013 [3]	22mo, F	Lateralized neck pain and torticollis	C1–2, extradual	Hypointense on T1w and T2w with minimal peripheral contrast peripheral enhancement	CD68(+). S100(+): a fragment of dorsal root ganglion was involved by the lesion in addition to a fragment of nerve root.	Debulking and biopsy	Non't mentioned
29	Song, 2015 [4]	77, F	Low back pain and both legs radicular pain 2 years	T12, extradual	Hypointense on T1w and T2w	HE: fibrous collagenesis with granular calcification	GT	No recurrence at 5 mo
30		67, F	Right leg radicular pain 2 months	L2–3, extradual	Calcification of the lesion on CT	HE: extratheloid cells in a granuloma-like pattern, fibrocellular stroma with spindled fibroblastic cells, and calcified materials	GT	No recurrence at 2 mo
31		78, F	Low back pain 2 months	L1, extradual	Calcification of the lesion on CT	HE: chondromyxoid matrix in a nodular pattern, palisading spindle to extrathelioid cells, fibrous stroma, calcification, osseous metaplasia, or scattered psammoma bodies, and foreign body type reaction with giant cell	GT	No recurrence at 1 mo
32	Reinard, 2015 [5]	44, M	Low back pain with left anterior thigh and left lateral calf radicular pain 3 years	L4, extradual	Hypointense on T1w and T2w with minimal contrast enhancement	EMA (+). The stromal component was non-reactive with CD34, a vascular marker.	GT	No recurrence at 4 years
33	Kocovski, 2015 [6]	64, F	Left low back pain with left leg radicular pain 6 months	L5-S1, extradual	Hypointense on T1w and T2w	HE: An extradural lesion with widespread amorphous and granular calcifying material and fibrous tissue	ST	No recurrence at 6 mo
34	Tao, 2015 [7]	56, M	Low back pain 1 year	L1, extradual	Hypointense on T1w and T2w, with minimal contrast enhancement	CD68(+),LCA(+),CD34(+), Desimin(−),EMA(−),Ki-67(1–10 %),PR9-,S-100(−), Vimentin(+), CK(−).	GT	No recurrence at 2 mo
35	Singh, 2016 [8]	90, F	Worsening lower extremity weakness 2–3 months	C7-T1, extradual	Isohypointense on T2w	HE: nodules composed of dense fibrous connective tissue with irregular layers of calcification, heavy central calcification, and a more cellular zone at the periphery of the nodule	ST	No recurrence at 2 mo
36	Lopes, 2016 [9]	72, F	Longstanding history of low back pain, cauda equina syndrome 20 days	L2,intradural	Hyperdense lesion on CT hypointense on T1w and T2w without contrast enhancement, no oedema	GFAP(+), EMA(+), SMA(+)	GT	No recurrence at 3 mo
37	Duque, 2016 [10]	51, F	Low back pain and both legs radicular pain 3 months	L2, extradual	Hypointense on T1w and T2w without contrast enhancement, no oedema	HE: extratheloid cells in a granuloma- like pattern, fibrocellular stroma with spindled fibroblastic cells, and calcified materials	GT	No recurrence at 3 years
38		46, F	Posterior neck pain 1 year	C3,intradural	Calcified intraosseous lesion on CT	HE: a typical chondromyxoid matrix in a nodular pattern with palisading spindles and extrathelioid and scattered psammoma bodies	GT	No recurrence at 2 years
39		73, M	Progressive paraparesis 6 month	T2, extradual	Hypointense on T1w and T2w	HE: a chondroid matrix with abundant fibrovascular stroma and a focal area of osseous metaplasia,	GT	No recurrence at 1 year
40	Giardinaet, 2016 [11]	68, M	Low back pain radiating into his right leg 6 month	L4–5,intradural	Isoipointense to the spinal cord on both T1w and T2w, without surrounding edema	HE: Spindle and extrathelioid cells bordered the chondromyxoid matrix nodules	GT	No recurrence at 5 years
41	Wu, 2017 [12]	39, F	Intermittent sacrococcygeal pain 17 years	S2,intradural	Calcification of the lesion and erosion of the vertebral arch on CT, hypointense on T1w, isohypointense on T2w with minimal contrast enhancement	HE: characteristic acellular chondromyxoidmatrix, including spindle extrathelial cells with calcium deposits and psammomatous bodies	GT	No recurrence at 3 years
42	Boschi, 2020 [13]	44, F	Back pain 2 months	T6–7, extradual	Hypointense on T1w and T2w	None	Indomethacin	No recurrence at 3 mo
43	Yang,2020 [14]	64, F	Left-sided neck pain 2 years following a fall	C2–4,extradual	Calcified nodules on CT and heterogeneous contrast enhancement on MRI	Neurofilament-light(+++)	GT	Stable at 2 years
44		60, M	Cervical myelopathy 3 weeks	C7,extradural	Isointense on T1w and T2w	Neurofilament-light(+++)	GT	Stable at 7 mos, improved myelopathy
45		51, F	Lower back pain 2 years	L3–4,extradural	Hypointense on T1w and T2w	Neurofilament-light(+++)	Debulking and biopsy	Stable at 2 mos
46		64, F	Lower back pain 6 months	L5-S1, extradural	Hypointense on T1w and signal void on T2w	Neurofilament-light(++), Neurofilament-phosphorylated(−)	GT	No recurrence at 6 mo
47	Lu, 2020 [15]	51, F	Lower back pain over 2 years	L3–4,extradural	Hypointense on T1w, mixed hypointense and hyperintense on T2w	HE: fibrous connective tissue containing granular amorphous or dystrophic calcified cores with palisading spindle to extrathelioid cells, fibrous stroma, and scattered CD68+ macrophages including occasional multinucleated giant cells	GT	No recurrence at 2 mo
48	Ho,2020 [16]	75, M	None	T11	None	NO classic chondromyxoid matrix, coarse and amorphous calcification, no surrounding meningothelial cells	None	None
49		52, M		T7–8	None	NO classic chondromyxoid matrix, coarse and amorphous calcification, surrongding meningothelial cells		
50		74, F		L5-S1	None	NO classic chondromyxoid matrix, coarse and amorphous calcification, surrongding no meningothelial cells		
51		68, F		L4–5	None	NO classic chondromyxoid matrix, coarse and amorphous calcification, surrongding no meningothelial cells		
52		49, M		L4–5, intradural	Hypointense on T1w and T2w	Classic chondromyxoid matrix, no coarse and amorphous calcification, no surrongding meningothelial cells		
53		43, F		T10–11, extradural	Hyperintense on T1w and hypointense on T2w	NO classic chondromyxoid matrix, coarse and amorphous calcification, surrongding meningothelial cells		
54		70, F		L4–5, transdural	Hypointense on T1w and hyperintense on T2w	NO classic chondromyxoid matrix, no coarse and amorphous calcification, surrongding meningothelial cells		
55		67, F		L4–5, extradural	Hyperintense on T1w and T2w	Classic chondromyxoid matrix, no coarse and amorphous calcification, surrongding meningothelial cells		
56		83, F		L3–4, extradural	Hypointense on T1w and hyperintense on T2w	Classic chondromyxoid matrix, coarse and amorphous calcification, surrongding meningothelial cells		
57		71, F		L5-S1,transdura	Hyperintense on T1w and T2w	NO classic chondromyxoid matrix, coarse and amorphous calcification, surrongding meningothelial cells		
58		50, F		L5, extradural	Hyperintense on T1w and hypointense on T2w	NO classic chondromyxoid matrix, coarse and amorphous calcification, surrongding meningothelial cells		
59		39, F		T9–10, Extradural	Hyperintense on T1w and hypointense on T2w	NO classic chondromyxoid matrix, coarse and amorphous calcification, surrongding meningothelial cells		
60		65, M		L2–3, extradural	Hyperintense on T1w and T2w	NO classic chondromyxoid matrix, coarse and amorphous calcification, surrongding meningothelial cells		
61		7, F		T2–3, extradural	Hypointense on T1w and T2w	NO classic chondromyxoid matrix, coarse and amorphous calcification, surrongding meningothelial cells		
62		78, M		T9–10, extradural	Hypointense on T1w and T2w	NO classic chondromyxoid matrix, coarse and amorphous calcification, surrongding meningothelial cells		
63		58, M		L2–3, intradural	Hyperintense on T1w and hypointense on T2w	Classic chondromyxoid matrix, no coarse and amorphous calcification, surrongding meningothelial cells		
64		77, M		C7-T1, extradural	Hyperintense on T1w and hypointense on T2w	NO classic chondromyxoid matrix, no coarse and amorphous calcification, surrongding meningothelial cells		
65		65, M		L3–4, transdural	Hypointense on T1w and on T2w	Classic chondromyxoid matrix, coarse and amorphous calcification, surrongding meningothelial cells		
66		71, F		L1–2, intradural	Hypointense on T1w and on T2w	Classic chondromyxoid matrix, no coarse and amorphous calcification, surrongding meningothelial cells		
67		86, M		Atlantooccipital, extradural	Hyperintense on T1w and on T2w	NO classic chondromyxoid matrix, coarse and amorphous calcification, surrongding meningothelial cells		
68		82, F		L4–5, extradural	Hyperintense on T1w and on T2w	NO classic chondromyxoid matrix, no coarse and amorphous calcification, surrongding meningothelial cells		
69		56, F		L4–5, transdural	Hyperintense on T1w and on T2w	NO classic chondromyxoid matrix, coarse and amorphous calcification, no surrongding meningothelial cells		
70		45, F		L4–5, extradural	Hyperintense on T1w and on T2w	NO classic chondromyxoid matrix, no coarse and amorphous calcification, no surrongding meningothelial cells		
71		43, M		T9-10, extradural	Hyperintense on T1w and on T2w	NO classic chondromyxoid matrix, coarse and amorphous calcification, surrongding no meningothelial cells		
72		52, F		C7-T1, extradural	Hypointense on T1w and on T2w	NO classic chondromyxoid matrix, coarse and amorphous calcification, no surrongding meningothelial cells		
73		64,M		C6	None	NO classic chondromyxoid matrix, coarse and amorphous calcification, surrongding meningothelial cells		
74	Ravi, 2021 [17]	53, F	Back pain with right leg radicular pain 3 years	L5,intradural	Isointense on T1w and T2w	HE: histiocytes and giant cells at the periphery of a chondromyxoid area.	GT	No recurrence at 6 mo
75	Lu,2022 [18]	64,F	Lateralized neck pain and torticollis 2 months	C3–4, extradual	Internal cystic components on T2w and heterogeneous enhancement on coronal T1 post-contrast enhanced	NF-L(+++), 7 CD8+ cells of 10 consecutive high-power fields in the most frequent positive cells	GT	No recurrence at 6 mo
76		60,M	Back pain with right leg radicular pain 1 years	L2-3, extradual	Isointense on T1w and T2w	NF-L(++), 37 CD8+ cells of 10 consecutive high-power fields in the most frequent positive cells	GT	No recurrence at 6 mo
77		71,F	Back pain 2 months	L2–4,intradural	Hypointense on T1w and T2w	NF-L(++), 25 CD8+ cells of 10 consecutive high-power fields in the most frequent positive cells	GT	No recurrence at 6 mo
78		51,F	Neck pain and left arm radicular pain 3 months	C3–5, extradual	Isointense on T1w and T2w	NF-L(+++), 181 CD8+ cells of 10 consecutive high-power fields in the most frequent positive cells	GT	No recurrence at 6 mo
79		64,F	Right leg radicular pain 3 months	L3-5, extradual	Hypointense on T1w and T2w	NF-L(++), 14 CD8+ cells of 10 consecutive high-power fields in the most frequent positive cells	GT	No recurrence at 6 mo
80	Omar,2024 [19]	35, M	Neck pain 6 months	C1, intradual extramedullary	Isointense on T1w and T2w	HE: peripheral cell palisading with multinucleatd giant cells	GT	No recurrence at 6 mo
81	Ajay,2024 [20]	66,F	Back pain and burning sensation in thigh	T11–12, intradual	Hypotense on T1w and T2w	HE: abundant hypocellular basophilic amorphous to fibrillated material with ghost cells, consistent with the characteristic chondromyxoid fibrillary matrix of CAPNON	GT	No recurrence at 6 weeks
82	Current cases	33,F	Back pain 5 years	L2-S1, intradual	Hypointense on T1w and mixed-signal on T2w	EMA(+). HE: Amorphous calcifying masses with osseous metaplasia and fibrovascular stroma in the lesion.	GT	No recurrence at 4 years
83		64,F	Lower limb numbness and walking unstable 2 moths	L3, intradural	hypointense on T1w and mixed-signal on T2w	EMA(+). HE: Tumor nodular growth with clear boundaries, visible calcification and ossification. Granular amorphous cores with calcification, peripheral palisading of spindle to extrathelioid cells, fibrous stroma and multinucleated giant cells.	GT	No recurrence at 1 year

Abbreviations CT, computed tomography; EMA, extrathelial membrane antigen; F, female; GFAP, glial fibrillary acidic protein; GT, gross total resection; M, male; mo, months; MRI, magnetic resonance imaging; SMA, smooth muscle actin; ST, subtotal resection; T1w, T1-weighted imaging; T2w, T2-weighted imaging; yrs., years.

Clinical presentation of radicular pain and sensorimotor deficits correlated with lesion location along dermatomal/myotomal distributions (Table 1). Patients were more likely to present with back pain (30 cases) than neck pain(11 cases) or leg pain(13 cases) (*p* = 0.0001), consistent with the lumbar predominance of these lesions. Notably, gait dysfunction occurred in one patient with a C3-C4 lesion and another with an L4-L5 level lesion, likely due to spinal cord or nerve root compression.

Imaging plays a crucial role in differentiating CAPNON from meningioma, oligodendroglioma, metastasis of calcified hematoma. CT typically reveals hyperdense calcifications. On MRI, lesions exhibit T2-weighted hypointensity due to calcification and may show variable enhancement, but lack typical meningioma features such as dural tails. Psammomatous meningiomas generally exhibit isointensity on T1 and heterogenous signal on T2, while metaplastic meningiomas show hypo-to isointensity on T1 or hypointensity on T2. Calcified meningiomas display perilesional edema more prominently than CAPNON. Additional entities like chordoma, chondrosarcoma and vestibular schwannoma typically appear as high signal on T2 with variable enhancement.

CAPNONs manifest as firm, well-circumscribed, and calcified lesions, which complicate surgical resection and necessitate prolonged operative times particularly for lengthy lesions such as case 1. Histopathology reveals a nodular chondromyxoid matrix with palisading spindles-to-epithelioid forms, fibrous stroma, psammoma bodies, and foreign-body reaction with giant cells. Focal EMA expression localized to arachnoid-related cells helps distinguish CAPNON from meningiomas, which show diffuse EMA positivity. The histologic variety supports a reactive etiology, possibly triggered by trauma, bacterial involvement, or inflammation. Meantime, the absent ribbon-like cells and vacuolated cytoplasmic textures renders chordomas unlikely. Finally, the lack of lymphocytes and Langhans giant cells excludes tuberculoma and bacterial pathogenesis. In our case, we report two positive focal EMA staining with one positive CD68 staining, which optimally established a consistent and steadfast immunohistochemical marker relevant to spinal CAPNON lesions.

Surgical excision remains the primary remedy, excluding two cases managed successfully with indomethacin 25 mg three times daily through inhibition of prostaglandin PGE2 which is an agent associated with heterotopic ossification processes. Misdiagnosis could lead to unnecessarily aggressive resection planned for chordomas, while CAPNON allows capsular preservation to avoid neurovascular injuries. Adjuvant therapies are ineffective. Gross total resection (38 cases) surpasses subtotal resection (13 cases) in frequency(*p* < 0.0001). Overall, an average follow-up time lasted approximately 12.4 months. Remarkably, our case study documented symptomatic relief upon discharge, adding to the large number of cases that indicate prompt ameliorative outcomes after operative CAPNON excisions.

## 4 Conclusions

Spinal CAPNON mostly located within the lumbar vertebrae, presented with low back pain and typically appeared T2-weighted hypointense on MRI sequences. Given that surgical excision is curative, differentiation from malignant tumors is required to avoid adjuvant therapy. Ongoing research is expected to further elucidate its underlying pathogenesis and refine optimal treatment strategies.

## Ethical approval

Ethics approval was obtained from the ethics committee of Changzheng Hospital (IRB number 2025-13).

## Guarantor

The guarantor (Zhang Dan-Feng) and all other authors agree with the work of the study to publish.

## Research registration number

No.

## Funding

The 10.13039/501100001809National Natural Science Foundation of China (82271396).

## Author contribution

Han Shuo: Writing-original draft, Supervision. Wang Guang-Ming: Conceptualization, Methodology. Dai Da-Wei: Supervision, Data curation and original data provision. Zhang Dan-Feng: Editing-original draft, Fund provision.

## Conflict of interest statement

No.
